# Supplementary material for: Smooth Interpolating Curves with Local Control and Monotone Alternating Curvature
Source: Comput Graph Forum. 2022 Oct 6;41(5):25–38. doi: 10.1111/cgf.14600 (PMC9827861; doi:10.1111/cgf.14600)
Supplement: Supplementary file 1 — Supplement Material [file CGF-41-25-s001.zip › Local-Smooth-Interpolating-MonoCurvature/extern/clothoids/docs/authors.html]

Authors — Clothoids v2.0.9

### Navigation

- index
- toc
- Clothoids »
- Authors

# Authors¶

- Enrico Bertolazzi

  Dipartimento di Ingegneria Industriale

  Università degli Studi di Trento

  email:

  homepage: https://e.bertolazzi.dii.unitn.it
- Marco Frego

  Faculty of Science and Technology

  Free University of Bozen-Bolzano

  email:

### Quick search

### Table of Contents

- Matlab Interface Manual
- C++ API
- MATLAB API

«
hide menu

menu
sidebar
»

### Navigation

- index
- toc
- Clothoids »
- Authors

© Copyright 2021, Enrico Bertolazzi and Marco Frego.
Created using Sphinx 4.2.0.
